# QSAR in the Browser: An Interactive Cheminformatics Web Application

**DOI:** 10.1021/acs.jcim.6c01010

**Published:** 2026-07-03

**Authors:** Syed Zayyan Masud, Theo Redfern-Nichols, Taufiq Rahman, Graham Ladds

**Affiliations:** Department of Pharmacology, 2152 University of Cambridge, Tennis Court Road, Cambridge CB2 1PD, U.K.

## Abstract

Cheminformatic analysis has been an active field for almost half a century, with considerable innovation accelerating drug discovery. However, the requirement for programming expertise prevents its popular use, often necessitating collaboration between multiple disciplines to integrate cheminformatics tasks into drug discovery pipelines. Various efforts have been made to mitigate this issue at the cost of cross-platform compatibility and preservation of data privacy. We introduce a static web application, QSAR, Quantitative Structure–Activity Relationship In The Browser (QITB), that performs various cheminformatic analyses on the user’s device, with no external server required. It includes tools to access the publicly available ChEMBL database and tools for users to upload their own data. It automatically processes data, offers a range of interactive tools for data visualization and analysis, and supports the training and evaluation of lightweight machine-learning models. By being hosted on GitHub Pages, the QITB web app is broadly accessible and enables the use of cheminformatics by experts and nonexperts alike.

## Graphical abstract

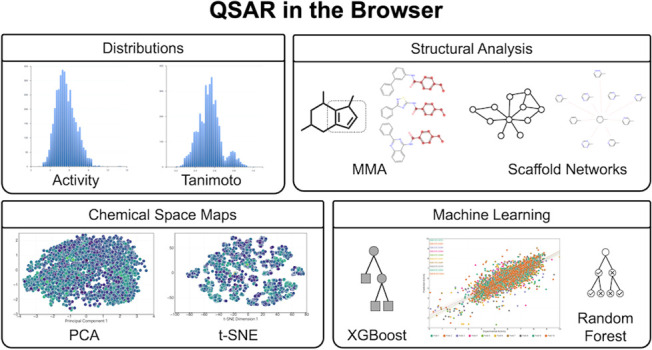

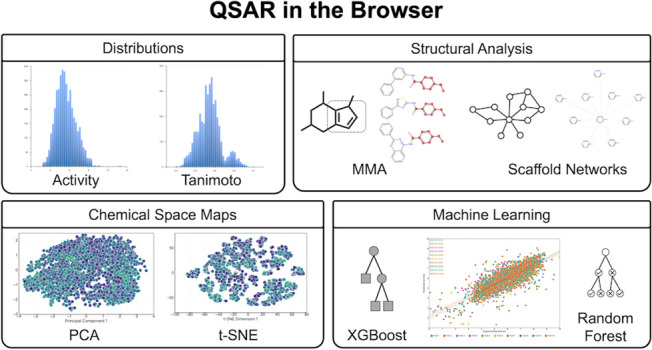

## Introduction

The relationship between a small molecule’s structure and its ability to affect biological targets has been studied for over half a century.^1,2^ The quantitative, statistical approach to analyzing these relationships is known as Quantitative Structure–Activity Relationships (QSAR). Even small structural changes can greatly impact ligands’ overall activity.^3^ Understanding this relationship facilitates the rational design and optimization of ligands, as seen with early nonpeptide angiotensin II receptor antagonists and selective serotonin (5-HT) receptor ligands.^4,5^

QSAR-based analysis is set to greatly benefit from the advancements in high-throughput data acquisition and the increased standardization of data formatting. These changes will likely broaden the relevance of QSAR and improve the predictive ability of QSAR methods incorporating machine learning (ML).^6^ However, the black-box nature and low interpretability of ML models necessitates a more detailed understanding of the small molecule data used to train them.^7^ Cheminformatic, QSAR-associated analyses and visualization techniques can provide this understanding, are well established, and have been used in the biological sciences for drug discovery for several decades.^8,9^

General QSAR analysis pipelines begin with data acquisition and handling. Only the chemical (2D/3D) structure of molecules and their performance metric(s) from suitable assay(s) are required. Freely accessible databases like ChEMBL provide large amounts of this kind of small molecule data. This allows QSAR workflows to work from publicly accessible data and not just private, proprietary collections.

Despite well-established techniques and available data, QSAR-related methodologies are not widespread. To utilize them efficiently requires investment in domain-specific expertise, computational competency or access to commercial software. Some applications overcome the requirement for technical expertise by combining QSAR workflows with Graphical User Interfaces (GUIs). However, as these application solutions often encompass entire drug discovery pipelines they can require installation, license registration or access to compute resources.^10–12^ Client-server applications allowing remote computation requires continued resourcing and support from the applications' developers to maintain the web servers.^13^ Hence, these solutions can still be limited by high licensing fees.

Serverless, static browser applications offer a potential solution due to their high accessibility and negligible maintenance costs, however this imposes strict computational constraints. Running QSAR analyses entirely within a browser restricts optimization strategies, memory usage and external library support, making advanced workflows challenging to implement.

Here we present QSAR-In-The-Browser (QITB) (https://qsar.syedzayyan.com), a fully client-side, static web application designed to enable nonexpert users to perform fundamental QSAR analysis. QITB can load bioactivity data directly from public resources such as ChEMBL, or operate entirely on user-supplied data sets that remain local to the device. The platform provides interactive visualizations and exploratory QSAR techniques to help users inspect chemical space and structure–activity trends. A key advantage of QITB is that it is delivered entirely as a static Web site: All computations occur within the browser with no external servers required. This serverless model maximizes accessibility, with design decisions intended to support efficient, responsive performance on standard consumer hardware. Being free and open-source, QITB also serves as an educational entry point into cheminformatics. Users can explore fundamental QSAR concepts without any application installation, coding experience or specialized infrastructure. Consequently, QITB lowers the barrier to cheminformatics by providing a fully browser-based, open-source environment that enables users to explore foundational QSAR concepts and prototype analyses without specialized software or computing resources.

## Implementation/Methods

QITB is freely accessible at https://qsar.syedzayyan.com with full source code hosted at https://github.com/syedzayyan/qsar-in-browser.

### Software Architecture

QITB is implemented as a client-side, static application, hence all computation is executed within the user’s browser, without reliance on external servers. The user experience involves an initialization phase where relevant data is locally loaded and processed (Figure S1A), followed by a data exploration phase (Figure S1B).

#### Frontend (NextJS Controller Layer)

The user interface (UI) is built using the NextJS framework, which enables prerendering into static HTML during build time. This approach enhances responsiveness and reinforces reproducibility across platforms. Additionally, NextJS’s component-based architecture simplifies the development of additional future UI features. The UI interacts with underlying computational components and tools through a controller layer (Figure S1A). The controller layer orchestrates the data loading, analyses requests and visualization updates (Figure S1B).

#### Backend Components

The QITB client-backend consists of computational tools structured from three main components: RDKit-MinimalLib (Custom Build) via WebAssembly (WASM); Pyodide (Python-in-WASM Environment) and WASM Wrapper (Figure S1B). This combination of tools allows high performance, quick calculation and some advanced features despite the limitation of in-browser implementation.

The RDKit-MinimalLib Custom Build provides cheminformatics functionality, parses Simplified Molecular Input Line Entry System (SMILES) strings, canonicalisation, molecule sanitisation and fingerprint generation. As this is compiled to WASM, near-native performance is achieved within the browser. The custom aspect enables extended functionality beyond the default distribution and has been submitted upstream as a pull request to the RDKit repository (PR #8920). Pyodide executes Python-based numerical routines including Principal Component Analysis (PCA), t-distributed Stochastic Neighbor Embedding (t-SNE), Random Forest training, eXtreme Gradient Boosting (XGBoost) training and k-fold evaluation.^14,15^ In QITB, Pyodide allows access to lightweight scientific Python packages, when JavaScript’s single-threaded model would be performance limiting. WebAssembly acts as the interface between Python, RDKit-MinimalLib and the NextJS controller (Figure S1B).^16–18^

#### Data Flow

QITB’s UI provides users three options for loading data into QITB: fetching data from ChEMBL (Figure S2A), uploading a user-supplied Comma-Separated Values (CSV) file (Figure S2B) or resuming a previously saved session (Figure S2C). Loaded data are then passed into the set of computational tools (WASM, RDKit and Pyodide). These computational tools then process data as results to be rendered in the frontend for interactive D3.js visualizations.

### Data Acquisition and Processing Pipeline

Users may search for any protein target listed in the ChEMBL database and use the associated small molecules for their QSAR analysis. QITB uses ChEMBL’s Web API to access the potential protein targets and fetch data.^19–21^ Once a target is selected, QITB displays two dropdown menus: “Assay Type” and “Unit Type”. These menus list the available options from ChEMBL’s database: available Assay Type categories include *binding*, *functional*, *ADMET*, *toxicity*, *physicochemical* or *unclassified*, while available Unit Type include *K*_*i*_, *IC50*, *XC50*, *EC50*, *AC50*, *K*_*d*_, *Potency* and *ED50* (Figure S2E,F). QITB provides the number of matching activity values for the Assay-Unit combination before fetching and loading the full data set, providing immediate feedback about data set size (Figure S2D). When the user confirms selection, QITB fetches a full activity data set for the specified protein target.

Alternatively, users may upload private ligand data sets via a CSV file (Figure S2B). The CSV file must include an ID column, containing string-type or numerical data, a SMILES string column and an Activity column, containing numerical data. These columns can have any name and are specified with QITB’s UI. Uploaded files remain entirely local due to QITB’s serverless architecture. Users may also save analysis sessions as a JavaScript Object Notation (JSON) file, and resume previous work sessions by loading these JSON files (Figure S2C).

All loaded SMILES strings are parsed and canonicalized using RDKit-MinimalLib. This ensures consistent and deterministic SMILES representation within RDKit. QITB’s default “Express” processing mode deduplicates molecules by ID and by RDKit-canonicalised SMILES and transforms data to a negative logarithm for appropriate plotting. In the “Advanced” processing mode, users can toggle data deduplication, and negative logarithm transformation for better graphical visualization.

QITB’s processing step additionally transforms ligand SMILES strings into molecular fingerprints using RDKit-MinimalLib. In the “Express” mode, ligands are converted to a 2048-bit Morgan fingerprint. In the “Advanced” mode, users can specify between Molecular ACCess System (MACCS), Morgan or RDKit fingerprints, and additionally specify additional parameters such as radius size and bit-length.^22,23^

### Data Visualization and Machine Learning Functionality

QITB supports exploratory and lightweight predictive QSAR analysis, implemented with Pyodide. Visual data exploration tools include activity distribution histograms, Tanimoto similarity distributions, PCA, t-SNE, scaffold enumeration and analysis via matched molecular series analysis and scaffold tree analysis.^24,25^ Lightweight predictive models include Random Forest regression or classification and XGBoost regression or classification. Both of which are implemented with k-fold evaluation support. These intentionally lightweight models are suitable for in-browser computation, providing pedagogical and exploratory value rather than production-grade predictive QSAR.

## Results

### Workflow Example–the Adenosine A_1_ Receptor

To demonstrate a typical workflow for QITB, we will use the publicly available ligand data for the Adenosine A_1_ Receptor (A_1_R). This prototypical G protein-coupled receptor (GPCR) is of particular pharmaceutical interest and of great interest to the authors.^26^ The dysfunction of the A_1_R is involved in a multitude of diseases.^27^ As such, a plethora of small-molecule ligands are available in the literature for the A_1_R.^28^ The ChEMBL database contains information about 4,000 unique ligands with associated *K*_i_ values representing the binding affinity to the A_1_R. After selecting the *Homo sapiens* A_1_R in QITB, 4,575 “Binding – *K*_i_” activity values are initially found. Applying “Express” processing with QITB yields 4,021 molecules (Figure 1A).

**Figure 1:**
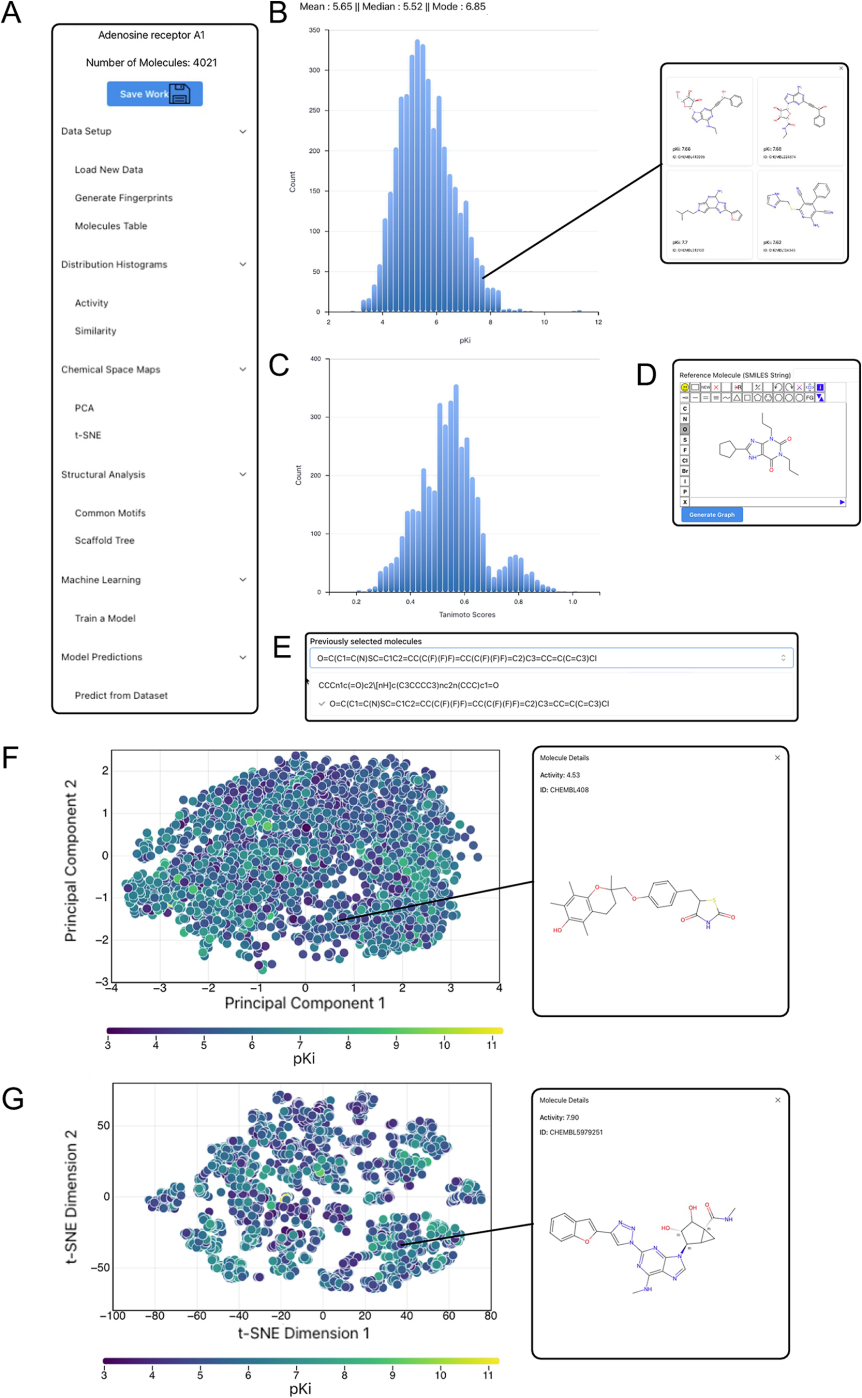
Data Visualizations Overview from QITB (A) The sidebar navigation menu showing chosen protein target, number of molecules, and ‘Save Work’ button, with every navigational item revealed. (B) The activity distribution histogram showing the total number of compounds within certain activity ranges. For this example, the activity measurement is the p*K*_i_ value of ligands targeting the A_1_R. Selecting a bar presents all molecules in that activity range in a popup card. (C) The Tanimoto distribution histogram showing the calculated Tanimoto scores for all compounds, based on their similarity to the A_1_R antagonist, DPCPX . (D) Integrated JSME having drawn DPCPX. (E) Dropdown menu to optionally show previous reference molecules for Tanimoto comparison. (F) PCA plot displaying each molecule as a point based on its calculated PC1 (*x*-axis) and PC2 (*y*-axis), the color of each point corresponds to its activity value, in this case, p*K*_i_ value of compounds targeting the A_1_R. The color bar below displays the mapping of the color to activity value. Selecting an individual point presents a pop up of that molecule with activity value, ID and 2D structure (G) A t-SNE plot using t-SNE-Dimension 1 and t-SNE-Dimension 2 as the *x*- and *y*- axes, respectively. Molecules are colored points using the same color mapping as the PCA plot. Selecting an individual point presents a pop up of that molecule with activity value, ID and 2D structure.

### Activity and Tanimoto Distributions

Following data processing, QITB presents an “activity” histogram (Figure 1B). This shows the distribution of the ligands across the selected activity value of interest. This histogram can be returned to at any time by selecting the “Activity” option under “Distribution Histograms”. In the A_1_R workflow example, this shows the distribution of p*K*_i_ values for the A_1_R. Above the chart, the mean, median and mode of the p*K*_i_ values of all ligands are provided. These parameters help to understand the average/most common activity values, which can be useful for identifying potential outliers or clusters of highly active or inactive compounds. Selecting a bar allows the user to see the molecules belonging to that data range.

Additionally, QITB allows users to visualize small-molecules similarity to a reference compound through Tanimoto distributions by selecting the “Similarity” option under “Distribution Histograms” (Figure 1C). The Tanimoto coefficient, when applied to molecular fingerprints, quantifies the structural similarity between two molecules where a score of 1 equates to a compound with an identical set of chemical features, while a score of 0 represents no chemical feature overlap. After providing a reference molecule, users can generate a Tanimoto distribution histogram. In the A_1_R workflow example, the data set is compared against 8-Cyclopentyl-1,3-dipropylxanthine (DPCPX), an antagonist for the A_1_R (Figure 1C). Users can provide reference molecules via a SMILES input box or by drawing molecules in the integrated JavaScript molecule editor (JSME) (Figure 1D).^29^ Previous reference molecules can be reselected using the provided dropdown menu (Figure 1E). Generally, for the A_1_R, ligand data Tanimoto scores are high when compared to DPCPX, potentially displaying two distinct populations of compounds, peaking around minor peak around 0.57 with 356 molecules and 0.79 with 64 molecules.

### Explore Data in an Advanced Data Table

In addition to providing a macro-level overview of the data, QITB allows users to precisely examine the data set using the “Explore Data” option under “Data Operations”. This feature offers a table showing all compounds, organized by columns of ChEMBL/user-provided ID, the SMILES string, a molecular diagram, and the activity value (Figure S3A). Physicochemical descriptors may be added for each molecule and dynamically presented with corresponding descriptions. Users can select individual molecules or several for removal from the data set. The data table also allows users to substructure search, finding molecules containing a particular substructure, using the SMILES input box or JSME.

### Chemical Space Maps

QITB can generate Chemical Space Maps which offer a less explicit but mathematically representative way to view the distribution of many compounds using dimensionality reduction methods. As high-dimensional vectors, molecular fingerprints are challenging to visualize, but dimensionality reduction methods offer a solution by projecting into fewer dimensions, making it suitable for visualization on 2D plots.^30^ QITB currently utilizes two dimensionality reduction algorithms: PCA (Figure 1F) and tSNE (Figure 1G). PCA is a linear, deterministic method that projects data onto orthogonal components capturing directions of maximum variance, while t-SNE is a nonlinear, stochastic method designed to preserve local neighborhood relationships and similarities between molecules.^31,32^

Users selecting “Chemical Space Maps” in the left menu bar are provided with options to view PCA or t-SNE Chemical Space Maps. Selecting “Run PCA” or “Run t-SNE” begins a background calculation of the respective chemical space map. This runs as a background process, allowing users to navigate QITB while the analysis proceeds. This optimization reduces crashes and improves the overall responsiveness of QITB. The user is notified when the calculation is complete and the page will present the PCA plot with PC1 and PC2 as the x and *y*-axes respectively (Figure 1F). Each point represents an individual small molecule in the data set and is colored based on the molecule’s activity value. Selecting “t-SNE” in the left menu presents the same options for t-SNE analysis and produces the equivalent plot using t-SNE-dimension 1 and t-SNE-dimension 2. Additionally, clicking the “t-SNE settings” dropdown arrow presents some t-SNE parameters. Selecting any point displays the 2D chemical structure, ID and activity value for the corresponding molecule.

### Structural Analysis

Users can analyze groups of molecules using QITB’s “Structural Analysis” tab. This is particularly useful for identifying underlying molecular scaffolds and potentially delineating the chemical factors that contribute to activity. Molecular scaffolds refer to generalized core structural frameworks that capture the key recurring features shared across related compounds. The precise definition of a scaffold can vary depending on the approach but the concept is widely used to group molecules by common structural motifs and to support interpretation and comparison of their properties. QITB offers two methods to analyze molecular scaffolds, “Common Motifs” and “Scaffold Tree”.

“Common Motifs” presents groups of molecules that share the same functional group on different scaffolds or share the same functional group on different positions of the same scaffold (Figure 2A).^33^ To allow interrogation of molecular moieties effects on activity values, QITB orders these groups according to their Kolmogorov statistic, which represents the statistical significance of changes in the activity value across the group.^33^ Selecting an individual group presents each compound in the group (Figure 2B), with the common functional group highlighted.

**Figure 2:**
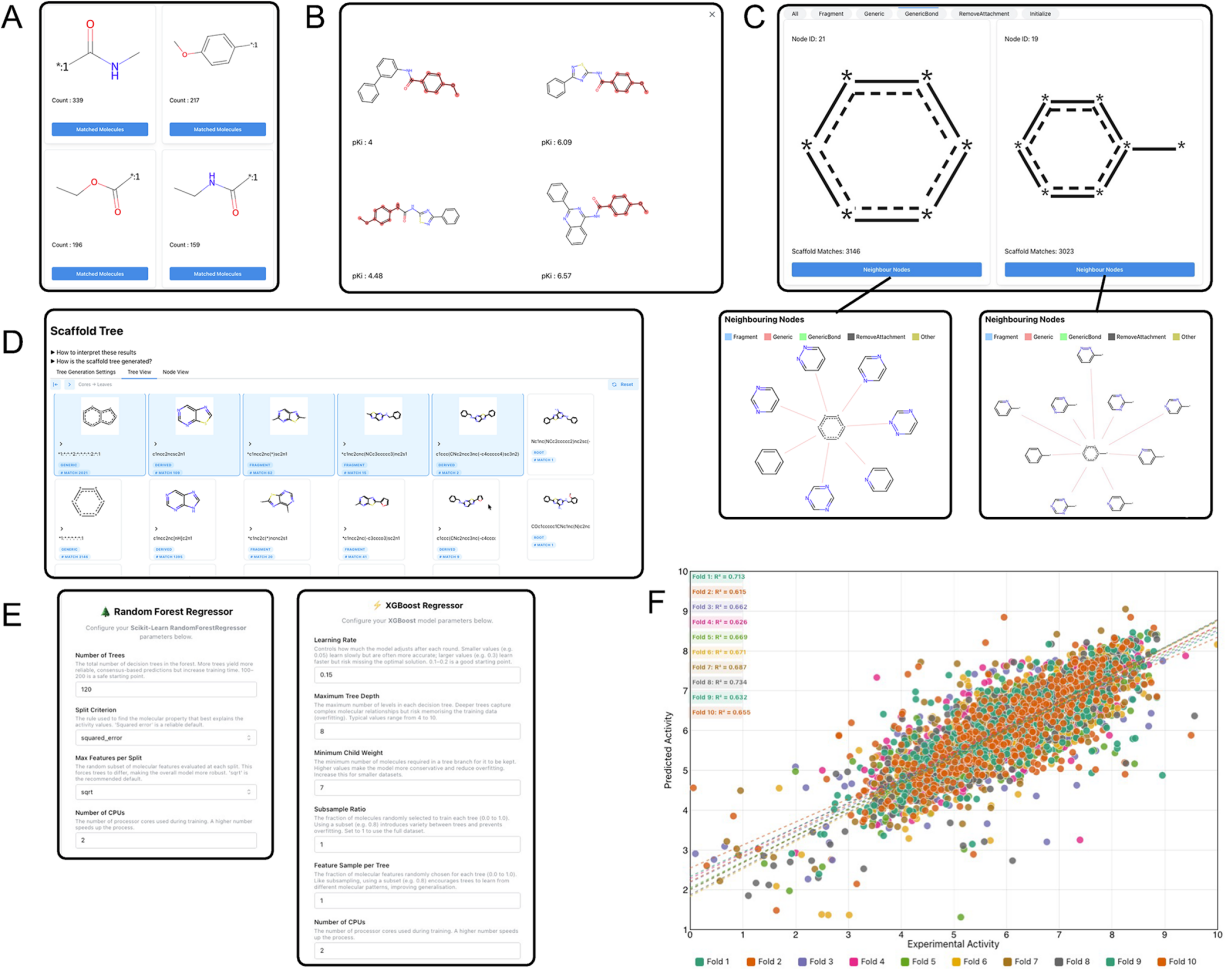
Scaffold Operations and Machine Learning. (A) The Common Motifs of the small molecule data set ranked by the number of “Matched Molecules”. (B) The collection of “Matched Molecules” presented after selecting a substructure, where the overlapping substructure is highlighted. (C) The “Node View” of a “Scaffold Tree” visualization, every node represents either a generalized scaffold, or at the root, an individual molecule. Selecting “Neighbour Nodes” reveals a local network of the more specific nodes belonging to that generalized node. (D) The Tree view of the Scaffold Tree visualization, selecting a node card reveals the less generic nodes that belong to it, which can be repeated until reaching a single molecule as the “Root Node” (E) The GUI and hyperparameter options available for training a Random Forest model on the ligand data set. (F) The performance for each fold of a 10-fold evaluation plotted as the model’s predicted values against actual experimental values. Each point denotes an individual molecule and its respective experimental activity (*x*-axis) and the model predicted activity (*y*-axis).

“Scaffold Tree” organizes the molecular data set into a hierarchical scaffold of components with more generic structures higher in the hierarchy and individual molecules at the lowest level.^24,25^ QITB presents two options for viewing (Figure 2C) this hierarchy, a “Node View” and a “Tree View”. The Node View displays every scaffold node as a card with filtering options to show different hierarchical levels (e.g., fragment, generic). Each card includes a “Neighbour Nodes” button which shows a local network of lower-level scaffolds derived from that node. Alternatively, the Tree View arranges all top-level generic structures in a single column, allowing users to progressively expand each branch to reveal increasingly specific subscaffolds until reaching a “Root Node”, corresponding to an individual molecule (Figure 2D).

### Machine Learning

Beyond visualizing and exploring small molecule data, QITB incorporates a set of lightweight ML tools designed to demonstrate how QSAR-orientated modeling pipelines operate in practice. Client-side, browser-based execution of ML tasks imposes strict computational limits. Despite this necessary limitation, QITB’s ML features include training, evaluation and prediction.

QITB’s currently supports two ML models; Random Forest, a widely used baseline method and XGBoost, a gradient-boosted tree-based approach.^34,35^ QITB also supports two training approaches, regression or classification. Regression is selected by default and involves training the model on continuous activity value data, in order for it to predict activity values. Users may also select a classification mode, where the small molecule data set is split into an active subset or inactive subset according to a user-defined activity threshold. Trained classification models output “active” or “inactive” for a given molecule. After selecting the training approach and model, the user is presented with hyperparameters with corresponding explanations. These are initially set at default values but can be adjusted as required by the user (Figure 2E).

By clicking “Train and Test”, QITB evaluates the model via 10-fold cross validation. This involves splitting the data into 10 equally sized parts—referred to as “folds”, training the model on 9 folds and evaluating its performance on the single, unseen, remaining fold. This is repeated, obtaining statistics for each fold. The results for each fold are then plotted on a parity plot comparing the experimental activity value (*x*-axis) and predicted activity value (*y*-axis) (Figure 2F). Additionally, users can view the Mean Absolute Error (MAE) across all folds with the bar chart (Figure S3D).

QITB also allows users to generate activity predictions using their trained model. Users may enter a molecule by providing a SMILES string or drawing the structure in the integrated JSME (Figure S3E). This same workflow can be applied for multiple molecules using the “Predict from Data set” option, which accepts a CSV file containing an ID column and a SMILES column (Figure S3F). In addition to user-supplied inputs, QITB provides an option to run predictions on the Broad Institute data set of bioactive molecules, allowing prediction of activity for a wide range of existing molecules.^36^ Prediction results are returned as a table sorted by the predicted activity values for each molecule (Figure S3G).

## Future Directions

QITB was designed as a browser-based platform with scope for continued extension in functionality and usability. The planned near-term development focuses on broadening data interoperability, expanding analytical options and improving the interpretability of outputs. Currently, user-supplied data sets are loaded through CSV files and public data access is centered on ChEMBL; future versions could support additional structure and file formats such as structure data format (SDF) and protein data format (PDB), alongside integration with other widely used public resources including PubChem,^37^ ZINC,^38^ DrugBank,^39^ BindingDB^40^ and GPCRdb.^41^

An important area for further development is the inclusion of applicability domain style guidance for the predictive models. This has excellent potential for demonstrating domain-specific training issues and assessing the ability for ML models to predict outside their training data set. For example, in addition to a predicted activity value, user-entered compounds could be contextualised by reporting their Tanimoto similarity to the training set. This would provide users with a simple indication of whether a prediction is being made within well-represented chemical space.

Developmental versions of QITB, available via the GitHub repository, include several extensions beyond those described in the stable release. These include additional molecular fingerprint options, more flexible data set-pruning operations, improved browsing of molecules associated with ML results and some early generative ML functionality.

Future releases may expand the set of available molecular representations and descriptors. In addition to the current fingerprint-based workflows, incorporation of a broader range of physicochemical descriptors could help users relate structural variation to more fundamental molecular properties and provide another level of interpretability during exploratory analysis.

There is potential support for selected neural-network-based QSAR methods using browser-compatible runtimes such as ONNX Web Runtime or TensorFlow.js.^42^ However, such developments must remain within the practical limits of in-browser execution, particularly memory availability and the need to maintain responsive performance on typical consumer hardware.

## Conclusions

The QITB web app performs complex cheminformatics tasks without the need for prior coding knowledge or any backend server. Hence the target audience for QITB is experts and nonexperts alike. The UI is built to be accessible to experts outside the realm of cheminformatics, with quick options existing for users new to cheminformatics and advanced settings available for those wanting more control. By making cheminformatics accessible, QITB can facilitate more research between groups of differing expertise, facilitating interdisciplinary science.

## Supplementary Material





## Data Availability

Data used in this article is from the publicly accessible ChEMBL database using their provided API.^20,21^ Readers can freely perform all analyses and create all featured graphs using the publicly available QITB web app, available at https://qsar.syedzayyan.com. The full source code, including the extended RDKit-MinimalLib build and processing scripts, is available at https://github.com/syedzayyan/qsar-in-browser.

## Notes

Graphic depicting QITB software architecture. Screenshots of QITB features not shown in the main figures, with clear instructions on how to replicate results in QITB (PDF)
Molecule data table extracted from the ChEMBL database by filtering for molecules with binding data for the human Adenosine A_1_ Receptor, used to create all figures shown in the article (CSV)

## References

[1] Hansch C., Maloney P. P., Fujita T., Muir R. M. (1962). Correlation of Biological
Activity of Phenoxyacetic Acids with Hammett Substituent Constants
and Partition Coefficients. Nature.

[2] Cherkasov A., Muratov E. N., Fourches D., Varnek A., Baskin I. I., Cronin M., Dearden J., Gramatica P., Martin Y. C., Todeschini R., Consonni V., Kuz’min V. E., Cramer R., Benigni R., Yang C., Rathman J., Terfloth L., Gasteiger J., Richard A., Tropsha A. (2014). QSAR Modeling:
Where Have You Been? Where Are You Going To?. J. Med. Chem..

[3] Koga H., Itoh A., Murayama S., Suzue S., Irikura T. (1980). Structure-Activity
Relationships of Antibacterial 6,7- and 7,8-Disubstituted 1-Alkyl-1,4-Dihydro-4-Oxoquinoline-3-Carboxylic
Acids. J. Med. Chem..

[4] Duncia J. V., Chiu A. T., Carini D. J., Gregory G. B., Johnson A. L., Price W. A., Wells G. J., Wong P. C., Calabrese J. C., Timmermans P. B. M. W. M. (1990). The Discovery of Potent Nonpeptide
Angiotensin II Receptor Antagonists: A New Class of Potent Antihypertensives. J. Med. Chem..

[5] Buckingham J., Glen R. C., Hill A. P., Hyde R. M., Martin G. R., Robertson A. D., Salmon J. A., Woollard P. M. (1995). Computer-Aided Design
and Synthesis of 5-Substituted Tryptamines and Their Pharmacology
at the 5-HT1D Receptor: Discovery of Compounds with Potential Anti-Migraine
Properties. J. Med. Chem..

[6] Li J., Zhao T., Yang Q., Du S., Xu L. (2025). A Review of
Quantitative Structure-Activity Relationship: The Development and
Current Status of Data Sets, Molecular Descriptors and Mathematical
Models. Chemom. Intell. Lab. Syst..

[7] Koirala M., Yan L., Mohamed Z., DiPaola M. (2025). AI-Integrated QSAR Modeling for Enhanced
Drug Discovery: From Classical Approaches to Deep Learning and Structural
Insight. Int. J. Mol. Sci..

[8] Neves B. J., Braga R. C., Melo-Filho C. C., Moreira-Filho J. T., Muratov E. N., Andrade C. H. (2018). QSAR-Based Virtual
Screening: Advances
and Applications in Drug Discovery. Front. Pharmacol..

[9] Clark D. E. (2006). What Has
Computer-Aided Molecular Design Ever Done for Drug Discovery?. Expert Opin. Drug Discovery.

[10] Flare QSAR models | Cresset. https://Cresset-group.com/software/flare-qsar-models/. (accessed July 28, 2025).

[11] Products BioSolveIT. BioSolveIT. https://www.biosolveit.de/products/. (accessed July 28, 2025).

[12] DeepAutoQSAR | Schrödinger Machine Learning Solutions.Schrödinger. https://www.schrodinger.com/platform/products/deepautoqsar/. (accessed July 28, 2025).

[13] Sicho M., Liu X., Svozil D., van Westen G. J. P. (2021). GenUI: Interactive and Extensible
Open Source Software Platform for de Novo Molecular Generation and
Cheminformatics. J. Cheminformatics.

[14] Droettboom, M. ; Hood, C. ; Yurchak, R. ; Dexter, C. ; Gyeongjae, C. ; Henry, S. ; Marc, A. ; Casatir; Will, L. ; Madhur, T. ; Max, M. J. ; Jason, S. Pyodide/Pyodide. 2021 10.5281/zenodo.5156931.

[15] TensorFlow.js | Machine Learning for JavaScript Developers.TensorFlow. https://www.tensorflow.org/js. (accessed July 28, 2025).

[16] RDKit. https://www.RDKit.org/. (accessed July 28, 2025).

[17] A new way to use the RDKit from other languages – RDKit blog.. https://greglandrum.github.io/rdkit-blog/posts/2021-05-01-rdkit-cffi-part1.html. (accessed July 28, 2025).

[18] Jiang C., Jin X., Dong Y., Chen M. (2016). Kekule.Js: An Open Source JavaScript
Chemoinformatics Toolkit. J. Chem. Inf. Model..

[19] ChEMBL Data Web Services | ChEMBL Interface Documentation. https://chembl.gitbook.io/chembl-interface-documentation/web-services/chembl-data-web-services. (accessed July 28, 2025).

[20] Zdrazil B., Felix E., Hunter F., Manners E. J., Blackshaw J., Corbett S., de Veij M., Ioannidis H., Lopez D. M., Mosquera J. F., Magarinos M. P., Bosc N., Arcila R., Kizilören T., Gaulton A., Bento A. P., Adasme M. F., Monecke P., Landrum G. A., Leach A. R. (2024). The ChEMBL Database in 2023: A Drug
Discovery Platform Spanning Multiple Bioactivity Data Types and Time
Periods. Nucleic Acids Res..

[21] Davies M., Nowotka M., Papadatos G., Dedman N., Gaulton A., Atkinson F., Bellis L., Overington J. P. (2015). ChEMBL
Web Services: Streamlining Access to Drug Discovery Data and Utilities. Nucleic Acids Res..

[22] Durant J. L., Leland B. A., Henry D. R., Nourse J. G. (2002). Reoptimization of
MDL Keys for Use in Drug Discovery. J. Chem.
Inf. Comput. Sci..

[23] Rogers D., Hahn M. (2010). Extended-Connectivity
Fingerprints. J. Chem.
Inf. Model..

[24] Varin T., Schuffenhauer A., Ertl P., Renner S. (2011). Mining for Bioactive
Scaffolds with Scaffold Networks: Improved Compound Set Enrichment
from Primary Screening Data. J. Chem. Inf. Model..

[25] Kruger F., Stiefl N., Landrum G. A. (2020). rdScaffoldNetwork:
The Scaffold Network
Implementation in RDKit. J. Chem. Inf. Model..

[26] Preti B., Suchankova A., Deganutti G., Leuenberger M., Barkan K., Manulak I., Huang X., Carvalho S., Ladds G., Lochner M. (2022). Discovery and Structure-Activity
Relationship Studies of Novel Adenosine A1 Receptor-Selective Agonists. J. Med. Chem..

[27] Wall M. J., Hill E., Huckstepp R., Barkan K., Deganutti G., Leuenberger M., Preti B., Winfield I., Carvalho S., Suchankova A., Wei H., Safitri D., Huang X., Imlach W., La Mache C., Dean E., Hume C., Hayward S., Oliver J., Zhao F.-Y., Spanswick D., Reynolds C. A., Lochner M., Ladds G., Frenguelli B. G. (2022). Selective
Activation of Gαob by an Adenosine A1 Receptor Agonist Elicits
Analgesia without Cardiorespiratory Depression. Nat. Commun..

[28] Matricon P., Nguyen A. T. N., Vo D. D., Baltos J.-A., Jaiteh M., Luttens A., Kampen S., Christopoulos A., Kihlberg J., May L. T., Carlsson J. (2023). Structure-Based Virtual
Screening Discovers Potent and Selective Adenosine A1 Receptor Antagonists. Eur. J. Med. Chem..

[29] Bienfait B., Ertl P. (2013). JSME: A Free Molecule Editor in JavaScript. J. Cheminf..

[30] Orlov A. A., Akhmetshin T. N., Horvath D., Marcou G., Varnek A. (2025). From High
Dimensions to Human Insight: Exploring Dimensionality Reduction for
Chemical Space Visualization. Mol. Inform..

[31] Maćkiewicz A., Ratajczak W. (1993). Principal Components Analysis (PCA). Comput. Geosci..

[32] van
der Maaten L., Hinton G. (2008). Visualizing Data Using T-SNE. J. Mach. Learn. Res..

[33] O’Boyle N. M., Boström J., Sayle R. A., Gill A. (2014). Using Matched
Molecular
Series as a Predictive Tool To Optimize Biological Activity. J. Med. Chem..

[34] Breiman L. (2001). Random Forests. Mach. Learn..

[35] Chen, T. ; Guestrin, C. In XGBoost: A Scalable Tree Boosting System, Proceedings of the 22nd ACM SIGKDD International Conference on Knowledge Discovery and Data Mining; KDD ’16; Association for Computing Machinery: New York, NY, USA, 2016; pp 785–794 10.1145/2939672.2939785.

[36] Corsello S. M., Bittker J. A., Liu Z., Gould J., McCarren P., Hirschman J. E., Johnston S. E., Vrcic A., Wong B., Khan M., Asiedu J., Narayan R., Mader C. C., Subramanian A., Golub T. R. (2017). The Drug Repurposing Hub: A next-Generation
Drug Library and Information Resource. Nat.
Med..

[37] Kim S., Chen J., Cheng T., Gindulyte A., He J., He S., Li Q., Shoemaker B. A., Thiessen P. A., Yu B., Zaslavsky L., Zhang J., Bolton E. E. (2025). PubChem 2025 Update. Nucleic Acids Res..

[38] Tingle B. I., Tang K. G., Castanon M., Gutierrez J. J., Khurelbaatar M., Dandarchuluun C., Moroz Y. S., Irwin J. J. (2023). ZINC-22–A
Free Multi-Billion-Scale Database of Tangible Compounds for Ligand
Discovery. J. Chem. Inf. Model..

[39] Knox C., Wilson M., Klinger C. M., Franklin M., Oler E., Wilson A., Pon A., Cox J., Chin N. E. Lucy., Strawbridge S. A., Garcia-Patino M., Kruger R., Sivakumaran A., Sanford S., Doshi R., Khetarpal N., Fatokun O., Doucet D., Zubkowski A., Rayat D. Y., Jackson H., Harford K., Anjum A., Zakir M., Wang F., Tian S., Lee B., Liigand J., Peters H., Wang R. Q. R., Nguyen T., So D., Sharp M., da Silva R., Gabriel C., Scantlebury J., Jasinski M., Ackerman D., Jewison T., Sajed T., Gautam V., Wishart D. S. (2024). DrugBank 6.0: The DrugBank Knowledgebase
for 2024. Nucleic Acids Res..

[40] Liu T., Hwang L., Burley S. K., Nitsche C. I., Southan C., Walters W. P., Gilson M. K. (2025). BindingDB in 2024: A FAIR Knowledgebase
of Protein-Small Molecule Binding Data. Nucleic
Acids Res..

[41] Pándy-Szekeres G., Caroli J., Mamyrbekov A., Kermani A. A., Keserű G. M., Kooistra A. J., Gloriam D. E. (2023). GPCRdb in 2023: State-Specific Structure
Models Using AlphaFold2 and New Ligand Resources. Nucleic Acids Res..

[42] Yang K., Swanson K., Jin W., Coley C., Eiden P., Gao H., Guzman-Perez A., Hopper T., Kelley B., Mathea M., Palmer A., Settels V., Jaakkola T., Jensen K., Barzilay R. (2019). Analyzing
Learned Molecular Representations for Property
Prediction. J. Chem. Inf. Model..

