# Supplementary material for: QSAR in the Browser: An Interactive Cheminformatics Web Application
Source: J Chem Inf Model. 2026 Jul 3;66(14):7805–12. doi: 10.1021/acs.jcim.6c01010 (PMC13417879; doi:10.1021/acs.jcim.6c01010)
Supplement: Supplementary file 1 [file ci6c01010_si_001.pdf]

QSAR in the browser: An interactive cheminformatics web application

Syed Zayyan Masud[1*], Theo Redfern-Nichols[1*], Taufiq Rahman[1] and Graham Ladds[1]

[1]Department of Pharmacology, University of Cambridge, Tennis Court Road, Cambridge, CB2 1PD, UK.

* These authors contributed equally.

Corresponding author: Prof Graham Ladds, Department of Pharmacology, University of Cambridge, Tennis Court Road, Cambridge, CB2 1PD Tel; +44 (0) 1223 334020. Email: grl30@cam.ac.uk

Supplementary information consisting of 3 supplementary figures.

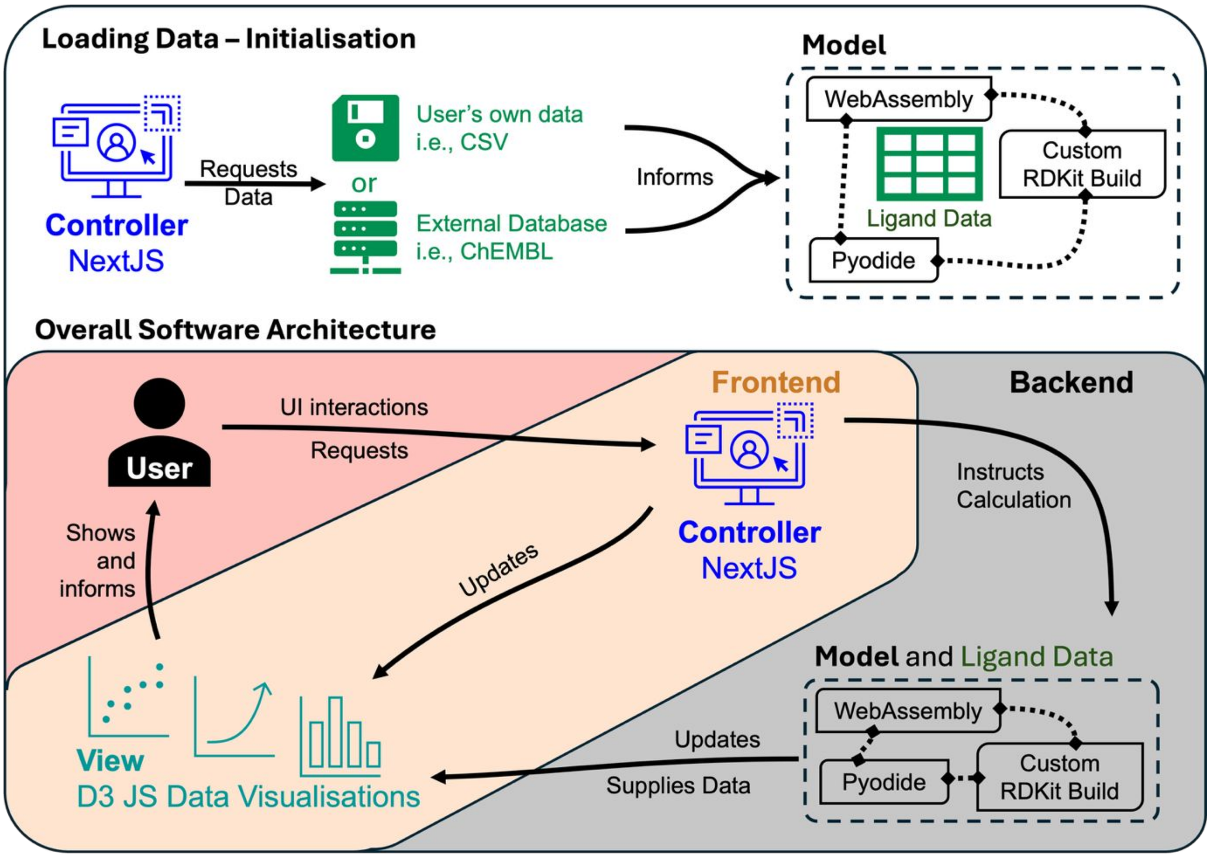

Figure S1 QITB's software architecture. (A) A central Controller (NextJS) module prompts data input from the user as either a user-supplied CSV file or an external data source such as ChEMBL. Both input streams feed into the application's Model layer. Within this layer, three computational components WebAssembly, a custom RDKit build, and Pyodide, operate in coordination based on the small molecule data. **(B)**. Once initialised, the user interacts with the frontend UI elements while being informed by frontend data visualisations. User interactions are received by the Controller which manages application logic and communicates with the backend computation. These backend calculations and model execute computational chemistry routines and data transformations. The backend then updates and supplies processed data back to the frontend View for rendering. The View subsequently shows and informs the user, completing the interaction loop.

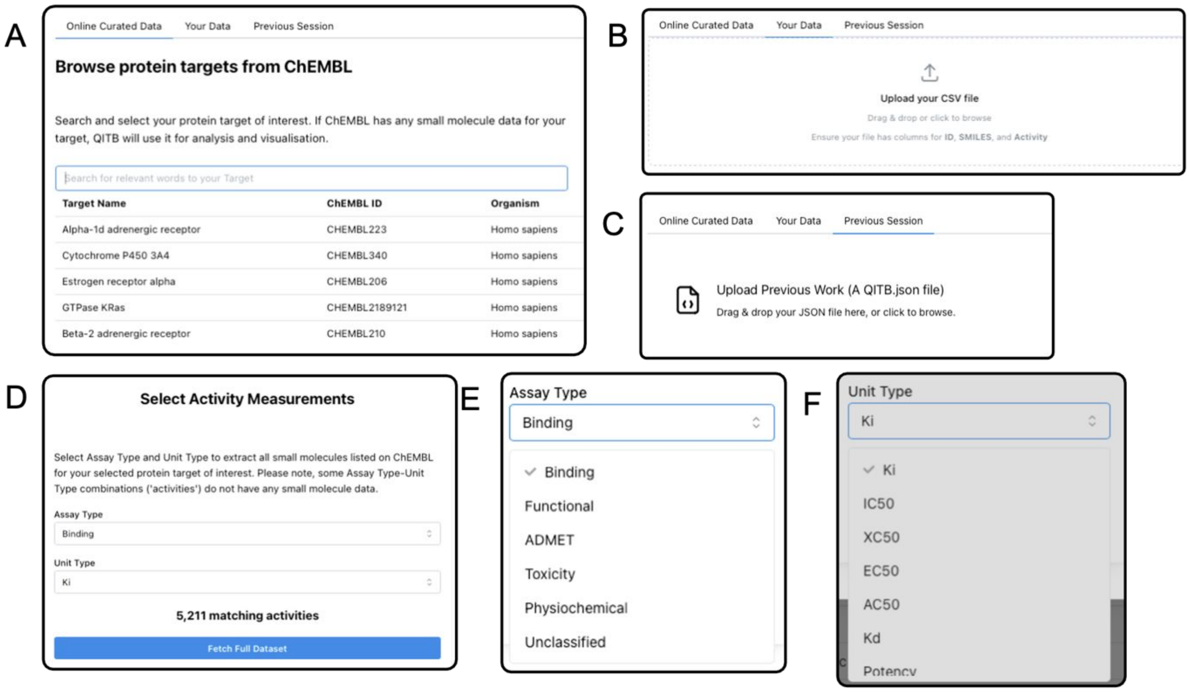

Figure S2 Data-loading UI elements. (A) The UI for data selection, specifically the 'Online Curated Data' tab showing how users can browse targets from the ChEMBL database. Users have two additional tab options for non-ChEMBL data selection: uploading local CSVs of data **(B)** or uploading a previous session in QITB, saved via json file **(C)**. **(D)** The dropdown menu to select 'Activity Measurements' after selecting a target protein, allowing choice of Assay Type **(E)** or Unit Type **(F)**.

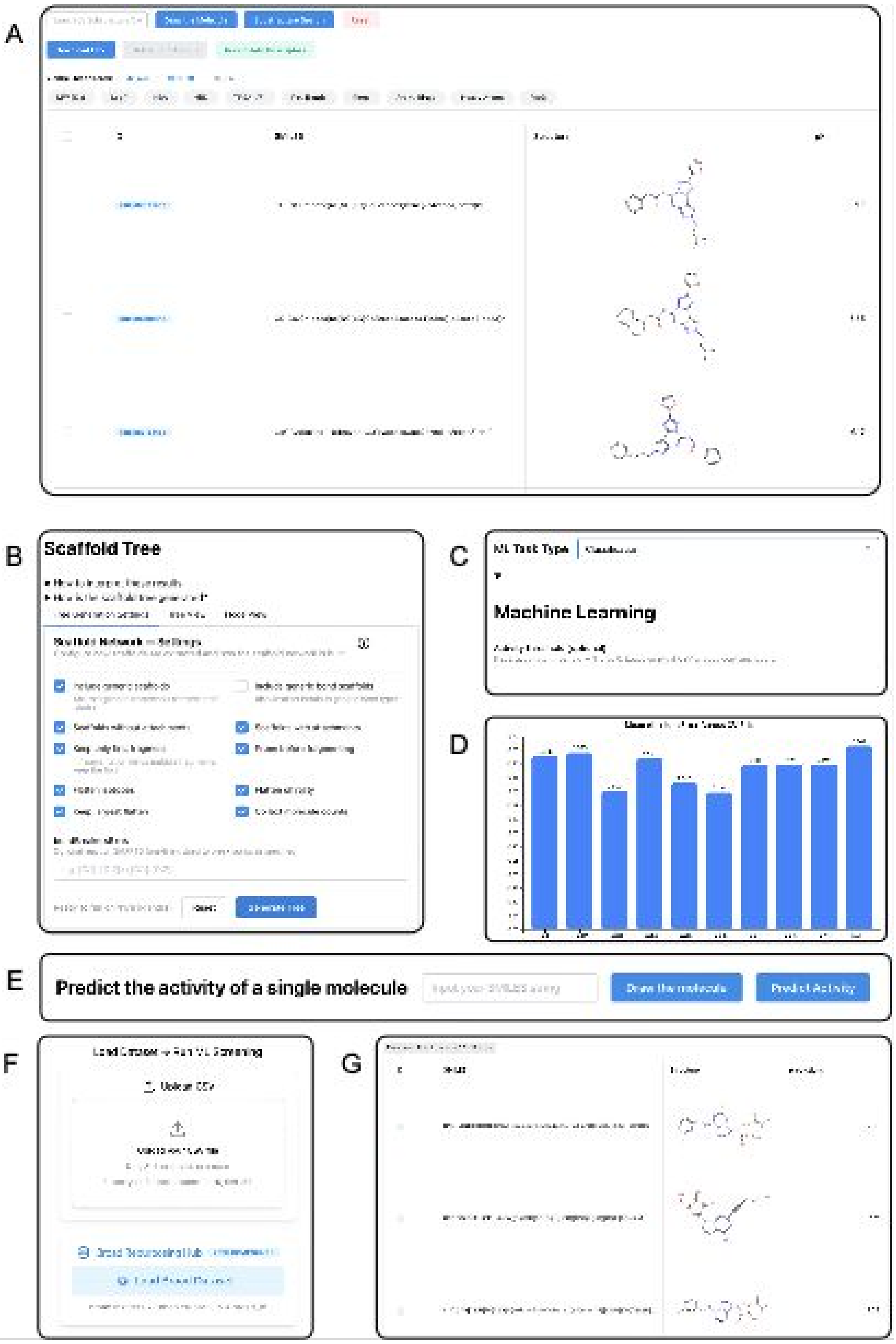

Figure S3 The Molecules Table, Scaffold Tree and Machine Learning features. (A) The Molecules Table with the ability to present physicochemical descriptors in additional columns. **(B)** The Scaffold Tree selection settings to customise the generation of molecular scaffold trees. **(C)** Classification is an available option in the Machine Learning (ML) 'Train a Model' section, allowing molecules to be labelled as active or inactive, based on a provided cutoff. **(D)** The Mean Absolute error for each fold of the k-fold evaluation, presented after model training. **(E)** The activity prediction

box available after model training, allowing prediction using the trained model. **(F)** The activity prediction interface for larger ML screens using CSVs or the Broad Institute dataset. **(G)** The ML screening results table displaying molecular structures alongside predicted activity values.
